# Stem cell biology and drug discovery

**DOI:** 10.1186/1741-7007-9-42

**Published:** 2011-06-07

**Authors:** Lee L Rubin, Kelly M Haston

**Affiliations:** 1 Dept of Stem Cell and Regenerative Biology and Harvard Stem Cell Institute, Harvard University, Cambridge MA 02138, USA

## Abstract

There are many reasons to be interested in stem cells, one of the most prominent being their potential use in finding better drugs to treat human disease. This article focuses on how this may be implemented. Recent advances in the production of reprogrammed adult cells and their regulated differentiation to disease-relevant cells are presented, and diseases that have been modeled using these methods are discussed. Remaining difficulties are highlighted, as are new therapeutic insights that have emerged.

## 

Stem cell biology has captured the imagination of biologists, tissue engineers, pharmaceutical company scientists, and indeed the general public, largely because of the prospect it seems to offer of manipulating cell fate to treat disorders for which there is no other effective therapy.

The initial focus was on diseases like type 1 diabetes and Parkinson's disease (PD), in which attempts had already been made to treat patients with donor cells [1,2]; but it was quickly recognized that embryonic stem cell (ESC) behavior may not be easy to control, and developing cells as safe and effective products is not as straightforward as developing small molecule or protein-based drugs, for which a great deal of experience has accumulated. The case of Geron, the biotechnology company that has been the first to initiate a clinical trial using ESC-derived cells, illustrates the hazards of developing a cell-based product [3]. This led to efforts to use stem cell biology to identify and develop small molecule drugs to target endogenous stem cell populations - for example, to stimulate neurogenesis to treat stroke, traumatic brain damage, Alzheimer's disease (AD) or PD, or other disorders of mood or cognition [4], or to inhibit stem cell-like cells in solid tumors [5].

In this review, we will focus on a variant of that idea: the use of human pluripotent cells in culture to produce differentiated cells that can be used as models on which to screen new drugs. One motivation for this is the widespread recognition that the drug discovery process as practiced in most pharmaceutical companies is inefficient, at best, and, in the past decade or so, has struggled to meet the need for new drugs. In addition, there have been a number of famous cases in which already marketed drugs have been found to have unanticipated side effects. Standard preclinical drug safety testing relies exclusively on administering drugs to two non-human animal species, and it is possible that safety studies on validated human cells might help avoid unexpected drug toxicities.

## Three key advances

From our perspective, the interest in stem cell biology as a route to novel therapeutic drugs arose from the convergence of three separate lines of investigation. First there is evidence that pathways that regulate embryonic development and, hence, act in large part on tissue stem and progenitor cells are also disrupted in adult disease [6,7]. For example, the hedgehog signaling pathway, of vital importance in nervous system development, is hyperactivated either by mutation or by ligand overexpression in a significant percentage of human cancers [8]. More than 10 years ago, we showed that it was possible to identify drug-like small molecules that inhibit hedgehog signaling and are effective in various cancer models [9,10], bringing together the worlds of developmental biology and conventional drug identification. In fact, as recently presented at the American Association for Cancer Research meeting by Dr Ervin H Epstein, a derivative of the first hedgehog antagonist developed, vismodegib, has been shown to have positive results in a phase II clinical study for metastatic basal cell carcinoma. Other hedgehog antagonists have already entered the clinic, including several developed by major pharmaceutical companies [11]. The observation that there is a link between stem cells, their regulatory pathways, and disease has clearly piqued the interest of the pharmaceutical industry, and there is serious interest in developing modulators of other pathways, such as Wnt and Notch, that are active in the embryo.

The second trend followed from a seminal discovery made by Jessell and co-workers [12] on the specification of motor neurons and other neurons in developing mouse spinal cord. They established a key role for sonic hedgehog-regulated signaling, and went on to show that the differentiation of motor neurons could be recapitulated in culture by adding retinoic acid to mouse ESCs to generate spinal cord progenitors and then an activator of the hedgehog pathway [12]. That was achieved with a small molecule that potently activates hedgehog signaling [13]. The lessons learned from this study were that: (a) it is possible, at least some of the time, to control differentiation of ESCs; (b) small molecules that regulate differentiation can be found; (c) by correctly controlling properties of stem and progenitor cells, it is possible to contemplate making large numbers of a defined type of cell. This work also opened up the possibility of making large numbers of differentiated cells from mice engineered to express human disease genes.

The third major advance was the reprogramming of adult cells to induced pluripotent stem cells (iPSCs), described by Yamanaka and co-workers [14,15]. The discovery that differentiated cells - for example, dermal fibroblasts - could be induced to revert to a pluripotent state made it possible to avoid both the political and the practical difficulty of using human ESCs, of which supplies are limited. iPSC technology offers the prospect of capturing cells derived from a large number of specific types of pre-diagnosed adult patients, potentially at any age, and a correspondingly large number of controls in a format that can support an industrial level of screening, efficacy, and safety studies.

## Stem cells as a tool for drug research and development

Stem cell biology is a rapidly growing field, and many excellent reviews of some of the topics covered here are available. In this article, we focus on using stem cells for drug research and development.

The central concept is that stem cells can provide a new means of studying the pathological basis of disease, screening for drug leads, testing candidate drug efficacy and safety, and selecting patient populations for clinical testing. The plan would be to identify a disease of interest and obtain skin biopsies or other tissue samples from patients with that disease. For each patient, iPSCs would be generated, expanded and (re)differentiated to the type of cells most affected in the disease of interest - for example, motor neurons for amyotrophic lateral sclerosis (ALS) or spinal muscular atrophy (SMA) - and to those most commonly affected by drug side effects (cardiac myocytes and hepatocytes). Once appropriate studies on disease mechanisms had been completed, screens could be set up to discover drug leads capable of correcting the disease phenotype. These screens may be phenotypic; for example, for ALS, a motor neuron survival screen could be appropriate. Hit compounds would be pursued by medicinal chemists (in the case of small molecule therapeutics) in the traditional way. But efficacy would continue to be tested on human diseased cells, and safety would be assessed in a preliminary fashion using corresponding cardiac and liver cells. Once potential lead compounds were identified, they would be tested on a broad sampling of individual patient-derived diseased cells, along with cardiac muscle and hepatocytes. This would aid in deciding whether certain compounds were more likely than others to be active across a large percentage of patients or, at a minimum, in preselecting the particular patients most likely to respond to a specific agent. The cost of drug discovery could be considerably reduced if a greater percentage of compounds entering the clinic were approved as drugs as a consequence of having better drug targets, better safety profiles, or a more considered choice of patient population.

How can we decide if this new approach can really evolve into an improved system of discovering and testing new drugs? Ultimately, the answer can only be provided in the clinic, and that will take a long time. However, prior to that, we will need to establish techniques to (a) produce patient-derived cells that are capable of multi-lineage differentiation; (b) regulate their differentiation into disease-relevant cell types; (c) use the differentiated cells to learn more about diseases of interest; (d) carry out primary screens and other types of efficacy testing on those cells; (e) assess a small number of the best compounds against a large sampling of patient-specific disease-relevant cells. These steps are explored in greater detail below.

## Producing cells with broad differentiation potential: iPSCs

The original methods of adult cell reprogramming were based on the use of viral vectors that drive the expression of the four transcription factors - Oct3/4, Sox2, c-Myc and Klf4 - identified by Yamanaka and colleagues. However, at least one of these - c-myc - has oncogenic potential [14,15], and these methods are also subject to the risk of insertional mutagenesis. This has led to efforts to produce iPSCs without genome modification. Most recently, a great deal of interest has surrounded a new method of reprogramming that is based on the addition of synthetic mRNAs encoding the four Yamanaka transcription factors [16].

At least some of the concerns associated with reprogramming would be avoided if it were possible to reprogram with just small molecules or proteins, and chemical biologists have also studied the reprogramming process. A large number of cocktails have been derived, all of which use different mixtures of small molecules and transduced genes (reviewed in [17]). The small molecules identified have typically replaced one or more of the reprogramming factors or have improved the efficiency of the overall process. Many of the screens have provided some insight into the mechanism of reprogramming. One example of such a screen was based on a simple experiment designed to identify a small molecule capable of replacing the transcription factor Sox2 [18]. Mouse embryo fibroblasts were transduced with retroviruses coding for Klf-4, Oct-4 and c-Myc, but not Sox2. Under those conditions, no true iPSC colonies formed. The cells were then treated with agents selected from an annotated compound library enriched in small molecules that modulate intracellular signaling. The most potent hit was an inhibitor of transforming growth factor (TGF)-β signaling. The surprise was in the way it acted: it increased expression of Nanog, another transcription factor with reprogramming activity. Furthermore, it affected not the starting cell population, but a population of partially reprogrammed intermediate cells that appeared 1 to 2 weeks after virus addition. This work, along with many other reports, demonstrates that reprogramming can be achieved in several, perhaps numerous, ways, with cells traversing different paths of dedifferentiation via many transient states of partial dedifferentiation. These reprogramming intermediates are, in a sense, artificial, being created as a result of an artificial process. This concept will be explored later in the context of regulating a real biological process: cell differentiation.

Disappointingly, at the time of writing, it has not been possible to reprogram cells completely with chemicals, although small molecules that replace individual transcription factors have been found. Is this because it is difficult to replace the activity of transcription factors effectively with small molecules? That may be true, although, as pointed out above, TGF-β inhibitors act by regulating the expression of a transcription factor. Is it because it is difficult to find combinations of small molecules that complement one another in the way that the transcription factors can? Is it just due to a lack of experimental insight into how best to replace these particular transcription factors? Answering these questions will be important because there are many other circumstances, reviewed later, in which small molecule modulation of cell fate could be valuable.

## How useful are iPSCs?

In the past few months, a spate of publications have highlighted problems that might be inextricably linked to reprogramming itself, perhaps independent of the particular method used [19-22] (reviewed in [23]). These include defects related to mutations, gene copy number variation, and incomplete resetting of DNA methylation. Some of these abnormalities may persist in differentiated cells produced from the iPSCs; some may be selected against by repetitive passaging. It is probably fair to say that iPSCs will be difficult to use therapeutically until these issues are resolved.

In spite of this significant concern, iPSCs may still have significant value in drug discovery. However, in that context, there is another potential problem. iPSC clones, even those prepared from a single patient, vary in their capacity to give rise to differentiated cells. Such variability has been seen previously with human ESC lines [24], which can show significant differences although they all meet the standard criteria for ESCs. That is, although they were all able to give rise to cells from the three germ layers *in vitro *and form teratomas in mice, some gave rise to endodermal lineages well, some gave rise to mesodermal lineages well, and so on. Thus, the standard criteria used to define pluripotency do not preclude line-to-line variability.

In an attempt to provide a systematic basis for characterizing stem cell lines, Bock and colleagues [25] carried out an extensive bioinformatics comparison of 20 human iPSC and 12 human ESC lines, including DNA methylation patterns, microarray analyses, and a general differentiation assay in which gene expression was analyzed in embryoid bodies derived from each line. On the basis of these data, it was possible to distinguish an average iPSC line from an average ESC line, although there was also considerable overlap. Importantly, the authors developed a scorecard based on a 500-gene expression array to quantify the differentiation tendencies of each line. The scorecard predicted that two of the iPSC lines might have reduced ability to differentiate into neurons and this was confirmed experimentally in a study carried out by Boulting and colleagues [26], who, however, also showed that most iPSC lines, whether derived from healthy controls of different ages and sexes or from different types of ALS patients, could be induced to differentiate adequately into motor neurons. This suggests that the variability of the cell lines may not preclude their use in screening. What remains to be measured is the degree of variability in cell response to therapeutic candidates. Do motor neurons produced from several different iPSC lines, all from the same patient, have the same response to potential drugs? Are data collected from motor neurons derived from different individuals reliable enough to predict clinical responsiveness across patients? Information like this is essential for the approach being discussed here. It will also be essential to develop methods for reliably inducing the various types of differentiated cells from stem cells. In that aim, there is good alignment between scientists interested in drug discovery and those focused on regenerative medicine (cell-based therapy). Thus, there is a real need to understand how to produce cells that are sufficiently differentiated to (a) model pathological aspects of disease; (b) faithfully predict drug safety; and (c) integrate effectively into tissue when transplanted.

## Embryonic development in a dish

**Figure 1:**
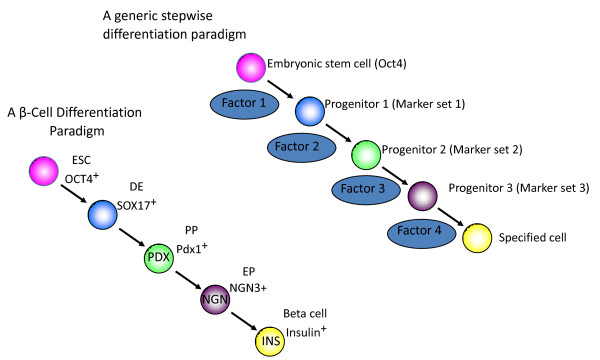
**The most common approach for regulating cell differentiation is based on coaxing cells through sequential stages of differentiation**. The top schematic is generic and could be applied to any cell type. The lower paradigm is one that could be used to produce pancreatic β-cells and is taken from the work of Chen *et al. *[43]. DE, definitive endoderm; EP, endocrine progenitor; PP, pancreatic progenitor.

Recent studies aimed at producing specific differentiated cells from ESCs or iPSCs have followed the principle established by Wichterle and colleagues [12] and attempted to recapitulate embryonic development in cell culture. At the core of this approach is the recognition that embryonic development occurs as a series of steps, with cells that have multipotential capacity becoming increasingly differentiated (Figure 1). However, even armed with this recognition, success has been somewhat mixed.

One instructive example is that of Kattman and colleagues [27], who published a very thorough paper describing a protocol to produce cardiac myocytes from ESCs and iPSCs in which they sequentially added morphogenic factors important in the appearance of cardiac muscle. They stressed a few general conclusions: (a) the first step of any differentiation procedure, the induction of the correct germ layer, must occur efficiently; (b) quantitative markers of different stages of development are helpful; (c) the timing of activation or inhibition of various morphogenic pathways is critical, especially given that the very same pathway can have a stimulatory or an inhibitory influence at different times; and (d) the concentration of the inducing factors must be controlled carefully. In essence, this work confirms that the complex environment of the embryo can be reproduced to at least some degree. However, the authors also pointed out that there is significant variation among different cell lines so that protocols may have to be tailored to each, perhaps because individual lines may make variable amounts of their own inducing factors. This would be a significant hurdle if it were necessary to produce cardiac myocytes from tens or hundreds of patient lines for drug toxicity testing. Thus, finding a way of overriding this variability would be a valuable advance.

Again by adopting an analogous strategy, Studer and colleagues [28] have pursued methods for producing particular types of neurons efficiently. Importantly, they introduced a convenient way of regulating early neural induction by treating human ESCs, grown without standard feeder layers, with inhibitors of both TGF-β and bone morphogenetic protein (BMP) signaling [28]. This group went on to show the utility of this technique in the generation of dopaminergic neurons and motor neurons. Subsequent studies confirmed its utility in the derivation of cell types as diverse as neural crest [29] and floor plate [30].

## Adding complexity to the culture environment

Eschenhagen and Zimmermann [31] have pointed out that the field of tissue engineering first arose as a consequence of efforts to produce functional tissue for implantation. Over the past few years, many investigators have tried to apply the principles of tissue engineering to the problem of producing individual types of differentiated cells by making the cell culture environment more like *in vivo *conditions, essentially by making it more complex. Vunjak-Novakovic and Scadden [32] have summarized the elements of a tissue engineering approach as including: (a) inducing factors; (b) extracellular matrix; (c) other cells, such as endothelial cells or stromal cells; and (d) physical factors, such as the rigidity of the tissue culture surface.

Various studies have incorporated some of these elements. As a simple start, numerous groups are interested in growing cells as three-dimensional aggregates, as a kind of intermediate between standard culture conditions and the true *in vivo *setting. In essence, both embryoid bodies and neurospheres are based on this philosophy. Mei and colleagues [33] published a very extensive study in which ESCs were plated on a combinatorial set of substrates and adsorbed proteins. They discovered a few combinations that supported ESC growth and colony formation particularly well. Approaches like this will undoubtedly prove useful, including as a way of replacing feeder layers or for encouraging uniform growth and spreading of cells across the culture surface. Underhill and Bhatia [34] described attempts to microfabricate extracellular matrix coated surfaces to allow cell growth, differentiation and survival. Several studies have emphasized the influence of the rigidity of the culture substrate. Gilbert and colleagues [35] found that muscle stem cells cultured on flexible hydrogel substrates like that found in real muscle retained more of their stem cell characteristics and performed better in a muscle regeneration assay. In another interesting application of tissue engineering principles, Domian and colleagues [36] induced differentiation of cardiac progenitors, purified them by fluorescence-activated cell sorting (FACS), and plated them on a fabricated thin film, thereby constructing a contractile sheet of cardiac muscle. These methods may turn out to be valuable in producing cells that are mature enough to adequately represent cellular function or dysfunction, as will be highlighted below.

## A transdifferentiation approach

**Figure 2:**
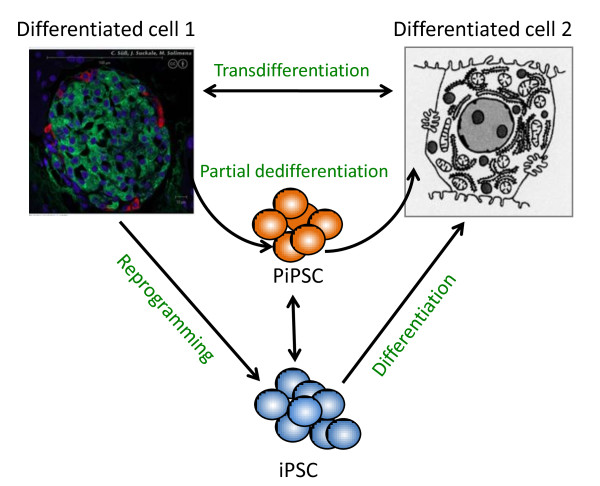
**Paths of cell differentiation**. There are many paths from one differentiated cell to another. These include reprogramming to an iPSC followed by differentiation, transdifferentiation from one differentiated cell to another, and partial dedifferentiation to a cell that we call a PiPSC (partially induced pluripotent stem cell) followed by differentiation.

A recent promising alternative way of producing differentiated cells, from large numbers of patients if necessary, is by direct reprogramming - or transdifferentiation - which is based on prior identification of transcription factors important in lineage specification (Figure 2). Just a few years ago, Zhou and colleagues [37] showed that pancreatic exocrine cells could be converted *in vivo *to pancreatic β-cells by infecting them with adenovirus expressing three transcription factors, *Ngn3*, *Pdx1 *and *Mafa*, all known to be important for β-cell development. Surprisingly, this occurred without proliferation of the exocrine cells, or even transient dedifferentiation to a progenitor cell state. Subsequently, Vierbuchen and colleagues [38] demonstrated that mouse fibroblasts, following treatment with lentivirus containing genes for three transcription factors expressed in the nervous system, *Ascl1*, *Brn2*, and *Myt1l*, could be induced to differentiate directly to neurons. Neurons could also be derived from glial cells, which are embryologically more similar, by expression of only *Neurog2*, a transcription factor important in neural determination [39]. Ieda and colleagues [40] showed that expressing three transcription factors important in heart development, *Gata4*, *Mef2c*, and *Tbx5*, could cause transdifferentiation of fibroblasts into cardiac myocytes.

These studies, and the profusion of those likely to follow in the near future, demonstrate fibroblasts need not be dedifferentiated completely to become other cell types. The potential advantage of this more direct approach, at least from a drug discovery perspective, is that more of the epigenetic modifications of patient-derived cells might be preserved if it were possible to bypass complete reprogramming. Also, there is some hope that this method may make it easier to produce more mature cells than one that relies on reversion of cells to a more embryonic cell-like state. Both of these differences could help in producing cells that more accurately model components of different diseases, although it is too early to judge how well the method will work. Can the fibroblasts be expanded sufficiently before viral transduction to allow for the generation of a sufficient number of differentiated cells? Can fibroblasts be obtained from older patients and still be transdifferentiated?

**Figure 3:**
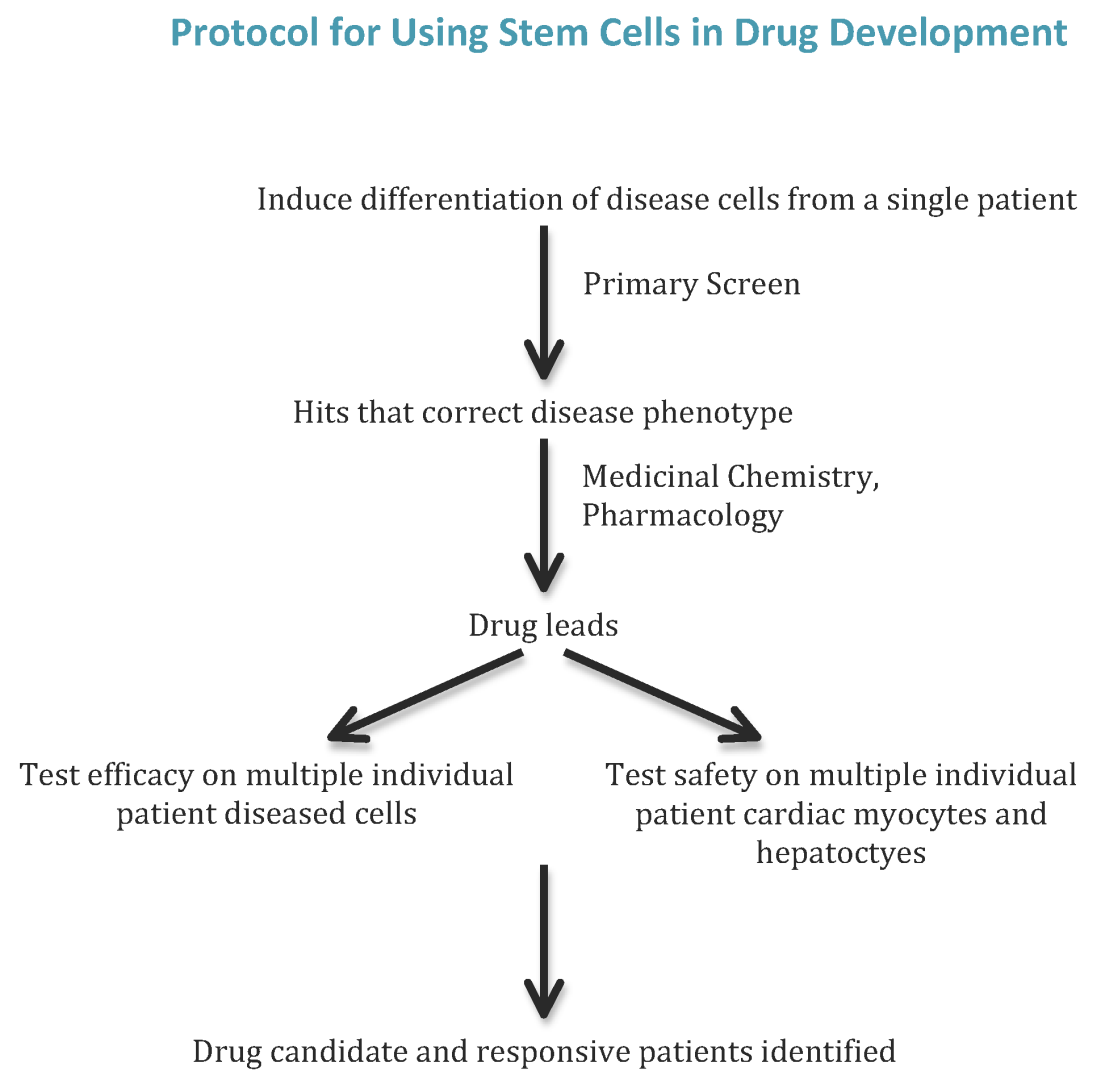
**A schematic diagram of a possible approach to using a stem cell-based system in a drug discovery campaign**. The central idea is that the cells used for screening, efficacy and safety testing would be patient-derived. Also, lines of cells, potentially prepared from many patients, would be used for efficacy and safety testing *in vitro *prior to testing on those patients *in vivo*. It is hoped that this will increase the probability of clinical success.

A third possibility has arisen that is based on partial dedifferentiation with a subsequent differentiation step (Figure 3). Reprogramming of mouse embryo fibroblasts is initiated but then aborted, and cells are put into a newly formulated medium that allows for the production of (in this case) cardiac myocytes [41]. Under certain circumstances, this method might allow for sufficient and rapid expansion of a type of progenitor cell still capable of multilineage differentiation.

## Small molecule regulators of differentiation

A final way of inducing cell differentiation is, in a sense, less rigidly adherent to the notion of replicating the precise inducing conditions that underlie *in vivo *development. The thinking behind this is that most investigators interested in therapeutics have the production of a single cell type as their main goal. That is quite different from what happens during development, where the production of many different types of cells must be regulated in a synchronized way in time and space. This raises the possibility that it may be simpler to produce certain types of cells (perhaps all types of cells) *in vitro *by manipulating cells in entirely different ways from those that operate *in vivo*. This would be consistent with the above-mentioned studies showing that cells can be reprogrammed by a wide range of combinations of genes and small molecules.

The question is whether this is true of differentiation as well, and if so, how could these pathways be identified? We and many others have adopted a screening approach in which stem or progenitor cells are treated with hundreds or thousands of small molecules and the effects on differentiation measured, generally by automated imaging. As reviewed previously [42], certain types of small molecule libraries are useful in these screens because, in principle, they allow for activation and inactivation of many different intracellular signaling cascades. On the basis of this idea, Chen and colleagues [43] followed the general format of sequential differentiation outlined in Figure 1, but, rather than restricting themselves to a small set of morphogens, tested about 5,000 small molecules in a successful effort to find agents that increase the production of pancreatic progenitors from human endodermal cells. An extensive analysis of marker gene expression showed that the chemically induced progenitor cells were highly similar to the ones that appear in the embryo and were capable of progressing further through development, producing a small number of functional β-cells.

The most effective small molecule hits in this screen were protein kinase C (PKC) activators. It will be interesting to determine if PKC activity is an essential part of early pancreatic differentiation *in vivo*. Alternatively, PKC might be a crucial component of an alternative path from endoderm to pancreas that is not used in the embryo. Thus, manipulation of certain receptors or signaling pathways may allow cells to escape their rigid developmental boundaries, while rendering them still capable of reaching a normal developmental endpoint. If this were true, it would have some far-reaching implications. First of all, it raises the question of how to establish whether the cells that are produced by the different methods are actually the same or at least similar enough. Recalling the discussion of pluripotency of ESCs and iPSCs, by many criteria, a variety of cell lines were classified as being pluripotent, but the standard criteria must have been too loose since the lines are variable. The same could be true with differentiated cells produced by different methods, so it may be necessary to use an equivalent of the scorecard described by Bock and colleagues [25]. Or, since there are, for the most part, two uses for differentiated cells - modeling disease *in vitro *and functioning appropriately when transplanted - it should be possible to establish practical criteria.

## Disease modeling using stem cells: can stem cells help us find better and safer drugs?

Assuming that issues with the production of pluripotent cells from patients' tissues, and the generation of differentiated cells from them, can be resolved, what other concerns remain? Unfortunately, there are many fundamental questions that have still not been addressed.

One important issue relates to the degree of maturity of the cells that are produced. For instance, ALS and SMA are both motor neuron diseases, but they affect different motor neuron populations. In ALS, neurons innervating distal muscles are most sensitive while, in SMA, those innervating proximal muscles are most at risk. Furthermore, in both diseases certain rare populations of motor neurons are completely unaffected so, in theory, when trying to model those diseases, using very specific types of motor neurons would be most appropriate. In fact, motor neurons produced by the most common differentiation protocols have a rather generic rostral cervical identity, although there is good reason to think that they can be induced to differentiate further by additional morphogens [44]. Presumably, a transdifferentiation approach in which the correct motor neuron pool-specific transcription factors are expressed in the motor neurons could also be successful.

This is not the only consideration, though. Typically, cells derived from pluripotent cells resemble their embryonic or immature counterparts (for example, [45]). Can they be induced to mature sufficiently to model adult disease? This has been hotly debated, especially in the context of late onset disease [46]. Many neurodegenerative disorders, such as AD and PD, take decades to affect humans and even many months to affect transgenic mice, so is it reasonable to think that neurons derived from stem cells could be induced to adopt a disease phenotype? It is possible that even in the late onset diseases, some of the pathological changes, such as protein aggregation, occur long before clinical symptoms. Another possibility relates to the fact that many of these diseases are primarily sporadic and may be initiated by the presence of particular environmental factors. Exposing cells to high concentrations of, or prolonged incubation with, these factors might greatly accelerate the appearance of pathology in the cell culture environment. For example, the addition of cellular stressors, such as pro-oxidants or other compounds that compromise mitochondrial function, might bring on disease-related alterations [45,46].

Another important issue concerns the nature of the diseases that realistically can be modeled by applying a reprogramming or even a transdifferentiation method to patient-derived cells. Naturally, monogenic diseases seem most amenable to this technique, and monogenic diseases that affect predominantly one cell type are likely to be better still. For these conditions, the expectation is that the reprogramming process will maintain the mutations involved, as will the differentiation protocol. However, what about diseases that are mostly sporadic and might involve epigenetic modifications of the genome? In those cases, reprogramming would tend to erase most of the epigenetic marks. Perhaps the transdifferentiation method will help in this regard, but this is not yet clear.

Certainly, the major degenerative diseases of the nervous system are primarily late onset and, while mostly sporadic in nature, are known to involve a small percentage of cases with well known disease-causing mutations. One way forward that may be both doable and instructive is to establish an *in vitro *phenotype using the genetic variants of the disease first, and then test the sporadic cases to determine if there are culture conditions that will produce the same disease pathology. Alternatively, it might be possible to identify pathology-producing cell culture manipulations that are informative about identifying the causative factors for the disease: for example, addition of certain insecticides may accelerate the onset of disease features in a PD model [45].

Starting just a few years ago, there have been many attempts to apply an overall stem cell strategy to the understanding of specific diseases. The typical starting point has been the production of patient-specific iPSCs. One of the first comprehensive reports was that of Park and colleagues [47], who derived them from patients with adenosine deaminase deficiency-related severe combined immunodeficiency, Shwachman-Bodian-Diamond syndrome, Gaucher disease type III, Duchenne and Becker muscular dystrophy, PD, Huntington's disease, juvenile-onset diabetes, and Down syndrome/trisomy 21. More recent efforts have included production of iPSCs from patients with SMA [48], ALS [49], and Hutchinson-Gilford Progeria Syndrome (premature aging, associated with vascular defects) [50,51]. A few illustrative cases will be presented.

## Nervous system disorders

One of the first examples in which an ESC-based approach (admittedly using mouse ESCs) contributed to a further understanding of disease mechanisms was that of ALS. Di Giorgio and colleagues [52] and Nagai and colleagues [53] established *in vitro *models of ALS by producing motor neurons from ESCs isolated from a transgenic mouse that carried a human superoxide dismutase mutation (G93A) found in a small percentage of patients with ALS. Although ALS is a late onset disease (decades in humans; approximately 4 months in mice), the authors, nonetheless, found a disease phenotype - death of G93A-expressing motor neurons was faster than that of wild-type motor neurons. In addition, they observed that astrocytes in the G93A motor neuron cultures appeared to secrete a toxic factor that further accelerated motor neuron death. The effect was selective in that interneurons were not killed by this factor. Subsequent work by Di Giorgio and colleagues [54] demonstrated that mutant mouse astrocyte-conditioned medium could also selectively kill human motor neurons produced from wild-type human ESCs. Thus, these investigators succeeded in modeling an adult-onset neurodegenerative disease and in gaining some insight into molecular mechanisms that underlie the disease. What remains uncertain is whether this conclusion can be generalized to other genetic forms of human ALS and whether it is possible to establish an informative disease phenotype starting with sporadic cases of ALS.

Another interesting recent study was carried out by Marchetto and colleagues [55]. Many neurobiologists have been interested in using an iPSC-based approach to study autism spectrum disorders (ASDs), a group of related neurodevelopmental defects. However, while there are undoubtedly genetic factors underlying these diseases, they are complex, and environmental factors seem to play a major role. These investigators chose instead to investigate patients with Rett syndrome, which is associated with impaired neural development about one year after birth and is caused by a mutation in the X-linked gene *MeCP-2*. Children afflicted with Rett syndrome have some of the symptoms found in other ASDs, but it is frequently used for this type of study because it is a genetic, rather than sporadic, disorder. This clearly makes it amenable to an iPSC type of approach. The group produced iPSCs from patients and from them prepared a mixed population of neurons, including GABA-ergic inhibitory neurons and glutamatergic excitatory neurons. Reassuringly, they found that the reprogramming process erased the X-inactivation of the *MeCP-2 *gene, but it was reestablished during neuronal differentiation, just as was hoped. Next, they found that there was not a large defect in survival of the induced neurons, at least after 2 months in culture. Nonetheless, there was a significant decease in the number of glutamatergic synapses, recapitulating the failure to appropriately form or maintain a normal number of functional mature synapses seen in the syndrome. Finally, they showed that insulin-like growth factor 1 (IGF-1), previously demonstrated to have some ameliorative effects in a mouse model of the disease, could increase synapse number in these human cultures. Thus, the authors demonstrated that the possibility of using an iPSC-based approach to gain an understanding of a complicated neural disorder and perhaps to screen for effective drugs.

Another good illustration of some of the points raised above is contained in a study on PD, a major neurodegenerative disorder affecting a subset of midbrain dopaminergic neurons [45]. Like ALS and AD, it is late-onset and mostly sporadic, although a set of disease-associated mutations has been identified, the most common of which is in the *Leucine-rich repeat kinase-2 *(*LRKK2*) gene. To model the disease, Nguyen and colleagues [45] derived iPSCs from patients with a *LRKK2 *mutation. They then followed standard protocols to produce neuronal cultures that were not pure, but did contain dopaminergic neurons that were physiologically active. By microarray analysis, the neurons were similar to those found in human fetal brain. Compared to neurons produced from control patient iPSCs, they had high levels of expression of oxidative stress genes. They also appeared to have a higher level of the protein α-synuclein, which forms characteristic aggregates in PD. Further, they seemed to be more susceptible to various stressors, such as hydrogen peroxide and 6-hydroxydopamine. Thus, although relatively immature, these iPS-derived neurons were capable of modeling some aspects of this late onset disease. Additional studies will be needed to see how completely they reproduce the disease phenotypes, how reproducible these changes are when larger numbers of iPSC lines are tested and how other types of PD patient-derived iPSCs will behave.

Finally, a study illustrating many of the points raised in this review was published by Lee and colleagues [56]. These investigators were interested in familial dysautonomia (FD), a genetic disorder associated with death of certain neural crest-derived neurons in sensory and autonomic ganglia. The disorder is associated with a mutation of the *IκB kinase complex-associated protein *(*IKBKAP*) gene, resulting in a splicing defect and reduced level of full-length IKAP protein. Fibroblasts were obtained from one young girl with FD and used to prepare iPSCs that were then induced to differentiate into different types of cells. Neural crest precursors showed a particularly low level of intact IKAP protein and had clear defects in migration and neuronal differentiation. Lee and colleagues further showed that kinetin, a plant hormone known to be effective when tested on lymphoblastoid cells from an FD patient, had some corrective effects on the FD neural crest precursors. This sets the stage for a more comprehensive drug screen using iPSC-derived cells.

## Cardiovascular disease and drug toxicity testing

Several interesting studies relate to cardiac myocytes made from reprogrammed cells. Itzhaki *et al. *[57] produced cardiac myocytes from iPSCs isolated from patients that have a K^+ ^channel mutation found in congenital long QT syndrome (LQTS), a disorder associated with cardiac arrhythmias. The myocytes also had increased action potential duration, and the authors were able to screen different pharmacological agents to see which ones could correct the underlying electrophysiological defect. In another study, Carvajal-Vergara and colleagues [58] pursued a very interesting and presumably rare disorder known as LEOPARD syndrome. It is characterized most frequently by hypertrophic cardiomyopathy and is caused by mutations in the gene (*PTPN11*) that codes for the phosphatase SHP2. Interestingly, iPSC-derived cardiac myocytes from patients were larger than those from controls, and the group has begun to dissect abnormal signaling within these cells that might be abrogated to ameliorate the disease phenotype. This is the kind of study, carried out in a rare disease background, that could contribute more generally to our understanding of other, more common types of cardiomyopathy.

The other widely discussed use for iPSC technology is in producing cardiac myocytes and hepatocytes to facilitate preclinical human testing of drug side effects. To date, much more progress has been made in producing cardiac cells. Braam and colleagues [59] carried out an early study using human ESCs as a source of human cardiac myocytes. They tested about 12 drugs that were already known either to affect or not to affect cardiac cells in patients. Similar activities were reproduced in the stem cell derived cultures. This is the beginning of toxicity studies that, in the future, should be done in the predictive sense: testing drugs on patient-derived cardiac cells before it is known how they will affect the patients. It will be especially important to decide how many patients' cells need to be tested to establish a sufficiently accurate estimate of the likelihood that an individual drug will have cardiac toxicity and, even more importantly, to help to identify biomarkers for sensitive and insensitive patients.

## Prospects for the future

There seems to be a widely held belief that the drug discovery system, as generally used in the pharmaceutical industry, needs to be improved, perhaps radically. In this article, we have suggested that a stem cell-based program might do just that by providing human disease-relevant cells in numbers large enough to be used to: discover new pathologies, thereby establishing better drug targets; carry out more predictive primary and secondary screening assays; test drug safety; and identify subsets of patients most likely to respond to particular therapeutic classes. This is summarized in Figure 3.

Much work needs to be done before we can be certain that differentiated cells produced from patient-derived iPSCs will offer any dramatic advantages. At present, we are uncertain whether the process of reprogramming, so essential to this method, is fundamentally flawed. Our view is that there will be many technical improvements over the next few years in the method of producing patient-specific differentiated cells (via reprogramming, transdifferentiation or partial reprogramming) to allow all of the relevant studies to be executed. A greater understanding will also be achieved with respect to the reproducibility of the process. At present, we do not know even how many iPSC clones per individual patient need to be produced to provide adequate consistency, nor do we know the true variability in response among cells derived from many patients with the same genetic disorder.

A final question that can be raised from the many types of *in vitro *studies described here relates to the seemingly ephemeral nature of the differentiated state. Why is it so relatively easy to change one type of cell into a radically different one? Does this suggest that cell identity could be changing, to at least some degree, much of the time? Does the existence of metaplastic cells also suggest the possibility that this phenomenon can occur without external manipulation? If so, does it further suggest the possibility that this process can be mobilized for therapeutic purposes? Will it be possible to interconvert cells using drugs - for instance, making muscle out of fat or connective tissue, or neurons out of glia - as an entirely different way of treating degenerative disorders or diseases of aging? If nothing else, new biological concepts derived from studying stem cell behavior may contribute to completely novel modes of treatment for serious diseases.
